# Serous adenocarcinoma of the fallopian tube, associated with verrocous carcinoma of the uterine cervix: a case report of synchronic rare gynecological tumors

**DOI:** 10.1186/1477-7819-7-20

**Published:** 2009-02-17

**Authors:** David Cantu de Leon, Delia Perez Montiel, Adan Tabarez, Rocio Mendez Martinez, Lucely Cetina

**Affiliations:** 1 Department of Pathology, Instituto Nacional de Cancerologia, Delegación Tlalpan, Mexico City, Mexico

## Abstract

**Background:**

Synchronous gynecological tumors are rare; it is even rarer to find the rarest of gynecological tumors that of the fallopian tube, together with a histological sub-type as rare as verrucous cervix.

**Case presentation:**

We report a synchronic fallopian tube adenocarcinoma and a verrucous cervical cancer. A 85-year-old woman with postmenopausal genital hemorrhage, endometrial biopsy was reported as squamous metaplasia, an exploratory laparotomy was performed finding a tubal tumor diagnosed as adenocarcinoma, a staging procedure was performed. Final staging revealed IB1 cervical carcinoma and IA G3 fallopian tube carcinoma. Adjuvant treatment with chemotherapy was not accepted by the patient. The patient has remained in follow-up, and at 9 months, there has been no documented evidence of recurrent disease.

**Conclusion:**

Reasons for our presentation of this work are: first, due to the rarity of these, and second, because of the usefulness of possessing a case report for establishing a norm for later behavior with respect to treatment of these patients.

## Background

Over the past years, an increase has been detected in the incidence of synchronous tumors, due mainly to the increment in life expectancy of the population and the development of ever more specific diagnostic methods that discover tumors that were not perceived previously [1], as well as improvement in the therapies employed, which ultimately afford time for the neoplastic lesion to develop in another organ. In the field of Gynecological Oncology, this type of lesion is infrequent, representing no more than 6% of cases [2].

The most common presentation is the combination of endometrium and ovarian neoplasms in the case of ovary, this is related with incessant ovulation, consequent estrogen production, and continuous stimulation of the endometrium, with the consequent formation of neoplasms characteristic of this site [2].

Malignant neoplasms of cervix, vagina, and vulva, in which the presence of the human papilloma virus (HPV) plays a very important role, comprise another example of synchronous tumors in which if the lesion is voluminous, it may not be possible to differentiate one from the other [3].

Association of the common risk factors in infrequent gynecological tumors such as those of fallopian tube, which represents < 2% of cases [4] is nearly impossible due to the reduced number of cases registered [1,4].

Cancer of the cervix is frequent neoplasm. The most frequently identified histologies are squamous, adenocarcarcinoma, and adenosquamous, these representing > 95% of cases, there are several rare histological variants of squamous carcinoma, such as verrucous, which is found at a much higher frequency in other sites such as oral cavity, skin, and larynx [5]. It possesses the characteristic of direct invasion to a greater degree than dissemination via lymph node pathway; thus, its treatment is, in general, surgical [6].

In this histological variant, the role of HPV has also been implicated as etiological agent. However, reports that exist in this respect have not been able to determine any HPV type in particular [7].

If synchronous gynecological tumors are rare, it is even rarer to find the rarest of gynecological tumors that of the fallopian tube, together with a histological sub-type as rare as verrucous cervix. We present a case of a patient with a primary carcinoma of the fallopian tube synchronous with a cervical carcinoma, verrucous type.

## Case presentation

A 84 year female was referred to the Instituto Nacional de Cancerlogía de México for abnormal postmenopausal genital hemorrhage. Family cancer history was negative. The patient reported no personal antecedents of importance related with the condition. On physical gynecological examination, there was no evidence of macroscopic lesions of vulva, vagina, or cervix; uterus was in 8-cm anteversion, and adnexa were not palpable. Colposcopy was performed, and this was reported as unsatisfactory due to an important atrophic cervical epithelium without evidence of acetowhite lesions and with normal vascular pattern.

A pelvic Ultrasound (US) was conducted reporting a 10-mm endometrial thickening. Fractionated endometrial biopsy showed condiloma. An exploratory laparotomy and total abdominal hysterectomy with bilateral salpingo-ophorectomy with frozen section study was planned. During the procedure, there was evidence of right adnexal tumor of 6 × 4 cm. A frozen section evaluation reported serous just carcinoma of right fallopian tube limited to fallopian tube; in cervix, a lesion in endocervical canal was identified, which was diagnosed as benign in the intraoperative pathological consultation; in uterus, there was no macroscopically identified endometrial lesion. Therefore, fallopian tube-staging surgery was completed.

Final staging was IB1 cervical carcinoma and IA G3 fallopian tube carcinoma according to FIGO staging system. The patient declined any adjuvant treatments, either chemotherapy or radiotherapy or both. The patient remained free of disease after nine months of close follow up.

### Pathology

**Figure 1:**
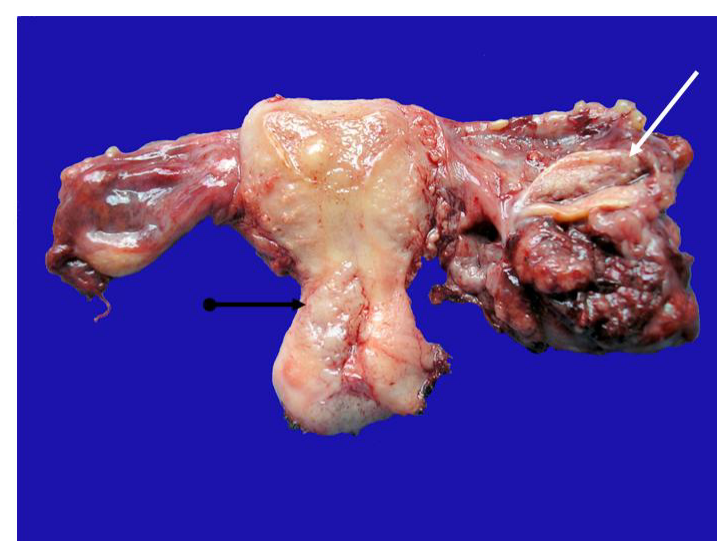
**The left salpinx shows a tumor mass with cauliflower like surface; the ovary is normal (white arrow)**. The endocervical wall is infiltrated by a neoplasia with a solid granular cut surface. (black arrow).

**Figure 2:**
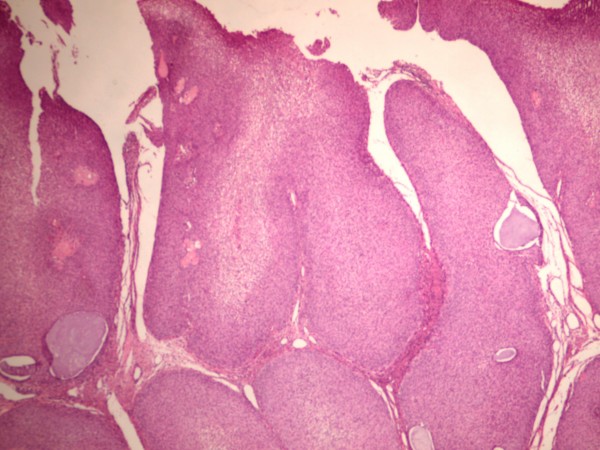
**Microscopic picture of the verrucous carcinoma of the cervix, the exophitic pattern is apparent (HE 4×)**.

Grossly the cervix showed a lesion in the endocervical canal very near the inferior segment that measured 2 × 1.1 cm, which partially obliterated the canal and infiltrated the cervical stroma on the left side. Right fallopian tube was dilated, and the lumen was occupied by a papillary-like neoplasm localized in the middle third of the fallopian tube, which extended to the external third (Fig 1). Ovaries, the left fallopian tube, and the uterine cavity exhibited no apparent lesions. Microscopic slides of the cervical lesion revealed that the tumor was constituted of a proliferation of squamous cells with slight atypia, koilocytic-like nuclei without alterations in maturation, and with scarce mitoses (1 × 20 fields high power); the borders of the neoplasm were thrusting and infiltrated 0.5 cm in a 1-cm cervical wall. (Fig 2) Vaginal border was negative for neoplastic cells. HPV typing of the cervical tumor was performed in order to investigate which virus was implicated, if any.

**Figure 3:**
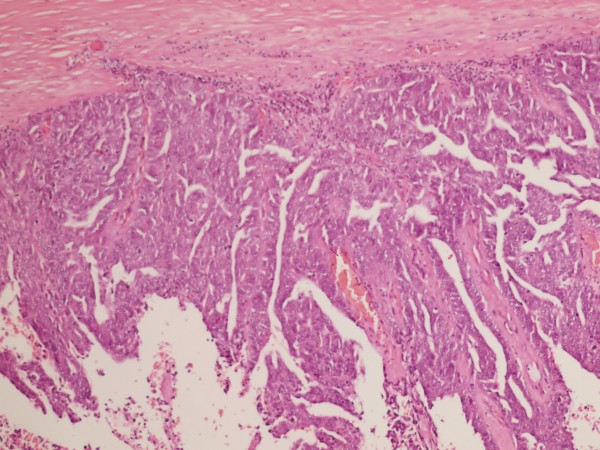
**Microscopic picture of salpinx tumor**. The neoplasm shows a papillary pattern with atypia in the cells (HE 10×).

The neoplasm of the fallopian tube was made up of papillae lined by a layer of cells with scarce cytoplasm, pleomorphic nuclei, with abundant atypical mitoses. The neoplasm infiltrated the fallopian-tube muscular wall without passing through the serosa (Fig 3).

### Molecular biology findings of verrucous carcinoma of the cervix

Fresh cervical tissue sample obtained during surgery was processed by means of the Quiagen DNeasy Tissue Kit for DNA extraction. Later, this was amplified with GP5+/GP6+ primers, which detects 35 types of HPV.

Positive PCR products were purified and subsequently sequenced in a programmable thermal cycler (Mastercycler gradient; Eppendorf^®^) using the BigDye Terminator v3.1

Cycle Sequencing Kit (Applied Biosystems) by using one of the PCR oligonucleotides as a sequencing primer(GP5+). The obtained sequence was compared with the GenBank database (National Center for Biotechnology Information, Bethesda, MD) by using the BLAST program resulting in that the sample was positive for type 11 HPV, no high risk HPV DNA was possible to identify [8].

## Discussion

At a specialized hospital such as the Instituto Nacional de Cancerología de México, the finding of synchronous tumors, despite their rarity (6%), is expected. Among rare gynecological tumors, tumors of the fallopian tube represents 2% [1-3,7,9,10]. Some authors consider a sub-registry of this tumor, because the clinical suspicion is not always available for identification of the pathological elements for differential diagnoses of tumor of the fallopian tube with ovarian epithelium [11].

The great majority of fallopian tube tumors are diagnosed after surgery by anatomopathological evaluation based on criteria established by Hu and modified by Sedlis as follows [11]: 1) If both fallopian tube and ovary are found to be involved, the greater tumoral burden should be found in fallopian tube; 2) Fallopian tube mucosa should be found involved and should show a papillary pattern, and 3) if the fallopian tube wall is found to be totally invaded, it should be possible to demonstrate the transition zone between benign and malignant epithelium.

Overall survival for cancer of the fallopian tube is 50–60% at 5 years; the majority of recurrences presents during the first 2–3 years, and are nearly all extrapelvic [10,11]. Initial treatment is surgical as in ovarian carcinoma. Adjuvant treatment with chemotherapy is with platinum- and taxane-based schemes, obtaining complete responses in up to 70% of cases [1,3-7,9,10]. Because there is no effective second line chemotherapy, prognosis after recurrence is ominous [10].

Verrucous carcinoma of the cervix represents less than 1% of cases [5], the relationship of verrucous carcinoma with HPV is suspected because the main site of the lesion is the outer labium of the cervix, which presupposes a sexual transmission pathway. Notwithstanding this, to date reports that exist have been unable to identify any virus serotype associated with this carcinoma [7]. In our case, we achieved identification of the human papilloma virus type 11, even though this viral type has been associated with benign lesions of the lower genital tract, some authors have associated low risk HPV [12] with the development of this specific variant of cervical carcinoma, other authors such as Frega et al in his review of three cases with the confirmed diagnosis of verrucous carcinoma all showed high risk HPV [13].

Treatment of choice is surgical, including treatment for recurrences in which total pelvic exenteration has to be considered; this is because invasion is due to a greater degree to local extension than to the lymph gland pathway. In general, this tumor type entertains a good prognosis when surgery is feasible. Radiotherapy should be avoided given that the tumor is radio-resistant and in addition, radiotherapy induced anaplastic changes that lead to regional and distant metastases as a consequence [5,7,9].

Recently there are some case reports of cervical and tubal carcinomas, one of them [14] was associated with two other tumors of the genital tract (endometrial and ovarian), the characteristic of this case is that both tubal and cervical carcinoma were from glandular origin while in our case cervical cancer was squamous in origin, the patient was staged properly and adjuvant treatment with chemotherapy was prescribed but she had pulmonary metastasis 15 months after initial treatment. Another case was reported by Ayas [15] were coexistence of an epidermoid carcinoma in situ of cervix was associated with a stage Ic serous papillary adenocarcinoma of the left fallopian tube, this case is interesting since is similar to ours in relation to the tubal neoplasm, patient was treated with adjuvant chemotherapy and is free of disease after 21 months, there are two differences with our case, the first and probably the most important is the nature of the cervical neoplasm which was preinvasive carcinoma in Ayas' case while in ours was an invasive rare variant of squamous carcinoma, and the other is adjuvant treatment, since our patient decided no to accept chemotherapy even though it is clear that chemotherapy which appears to improve the efficacy of surgery, both cases are free of tumor on follow-up. Age in both patients is not similar; our patient is in the ninth decade of life which is in the range of age for the tumor while the other is just 39 years old.

Several reports show relation between abnormalities on cervical smears and tubal carcinoma, these abnormalities are characteristically glandular rather than squamous and is important to mention that no association with human papilloma virus induced abnormalities had been shown [16].

## Conclusion

We can conclude that synchronous gynecological tumors are rare, most frequent association of fallopian tube tumors is with endometrium, although there are reports that support their relationship with breast cancer. Verrucous carcinoma is very rare in cervix, entertains a good prognosis, and is a type of cervical cancer in which radiotherapy results in damage rather than in benefit. Reasons for our presentation of this work are: first, due to the rarity of these, and second, because of the usefulness of possessing a case report for establishing a norm for later behavior with respect to treatment of these patients.

## Consent

Written informed consent was obtained from the patient for publication of this case report and any accompanying images. A copy of the written consent is available for review by the Editor-in-Chief of this journal.

## Competing interests

The authors declare that they have no competing interests.

## Authors' contributions

DCL was involved in the design and writing of the manuscript. DPM was involved in pathologic evaluation and writing of the manuscript. AT was involved in literature and case review. RMM was involved HPV detection typing and genetic sequence. LC was involved in manuscript completion and critical review. All authors read and approved the final manuscript.
